# The causal influence of responsive parenting behaviour on academic readiness: a protocol for a systematic review and meta-analysis of randomized controlled trials

**DOI:** 10.1186/s13643-021-01757-8

**Published:** 2021-07-20

**Authors:** Heather Prime, Krysta Andrews, Andrea Gonzalez, Magdalena Janus, Andrea C. Tricco, Teresa Bennett, Leslie Atkinson

**Affiliations:** 1 Department of Psychology, York University, Toronto, Canada; 2 LaMarsh Centre for Child and Youth Research, York University, Toronto, Canada; 3 Department of Psychiatry and Behavioural Neurosciences, McMaster University, Hamilton, Canada; 4 Offord Centre for Child Studies, Hamilton, Canada; 5 Knowledge Translation Program, Li Ka Shing Knowledge Institute, St. Michael’s Hospital, Toronto, Canada; 6 Epidemiology Division, Dalla Lana School of Public Health and Institute for Health, Management, and Evaluation, University of Toronto, Toronto, Canada; 7 Queen’s Collaboration for Health Care Quality, Joanna Briggs Institute Centre of Excellence, School of Nursing, Queen’s University, Kingston, Canada; 8 Department of Psychology, Ryerson University, Toronto, Canada

**Keywords:** Responsive parenting, Academic readiness, Randomized controlled trials, Mediating processes, Causal process

## Abstract

**Background:**

Children’s academic readiness has important implications for subsequent achievement and psychosocial functioning. A growing number of studies are utilizing randomized controlled trials (RCT) to examine whether responsive parenting interventions lead to positive gains in children’s academic readiness. A synthesis of the extant literature is warranted to gain a precise estimate of the causal influence of responsive parenting on academic readiness, as well as to examine moderators that may serve to strengthen or weaken this effect. The main objective of this study will be to conduct a systematic review and meta-analysis of RCTs evaluating the use of responsive parenting interventions to target academic readiness: problem-solving/reasoning, language proficiency, executive functioning, and pre-academic skills (e.g., numeracy/literacy).

**Methods:**

Studies that took place in the early childhood period (< 6 years at baseline), targeted responsive parenting behaviours using an RCT (with control group, waitlist, or treatment as usual as a comparator), and included an outcome assessment of academic readiness will be considered for eligibility. Children and/or parents with special needs and/or disabilities will be excluded. The primary outcome is the effect of responsive parenting interventions on academic readiness. Secondary outcomes include substantive and methodological moderators and parent-mediated effects on outcomes. We will search MEDLINE, PsycINFO, ERIC, and ProQuest Dissertations & Theses Global databases from their inception onwards and we will also conduct backward/forward searching of eligible studies. Published and unpublished works will be considered. Screening, full-text assessments, and data extraction will be completed by two independent reviewers. Risk of bias will be assessed using the CLARITY tool for RCTs. Effect sizes will be calculated based on study-level standardized differences between experimental and control groups and entered into random effects models to obtain a pooled effect (meta-analysis). Moderation will be examined through *Q-*statistics and meta-regression to study sources of between-study variation in effect sizes. A pooled path model of mediation will be used to study parent-mediated effects.

**Discussion:**

Findings will illuminate causal relations between responsive parenting and academic readiness, with implications for developmental science. Findings will also guide decision making in policy and practice for supporting early childhood development and reducing social disparities in children prior to school-entry.

**Systematic review registration:**

PROSPERO CRD42020222143.

**Supplementary Information:**

The online version contains supplementary material available at 10.1186/s13643-021-01757-8.

## Background

Children’s developmental health includes skills, states, and competencies across physical health and development, social-emotional well-being, and academic readiness and is largely influenced by children’s early experiences [1]. Amongst these indicators of developmental health, academic readiness reflects a subset of skills at school entry, including problem-solving/reasoning, language proficiency, executive functioning, and pre-academic skills (e.g., numeracy/literacy). It is considered foundational to long-term outcomes, including psychosocial functioning and academic success [2, 3]. As such, efforts to develop, evaluate, and disseminate programmes to support academic readiness across the infancy and early childhood period have been prioritized in recent years.

As the primary proximal influence in children’s early environments, parenting is a commonly examined correlate of individual differences in academic readiness. Specifically, responsive parenting behaviours, both emotional-affective (e.g., warmth and sensitivity) and cognitive in nature (e.g., stimulation, maintaining interests), have been longitudinally linked to the emergence of academic readiness [4, 5]. To date, the synthesis of research examining links between optimal parenting in early childhood and children’s academic readiness comes from naturalistic observational studies, including in the areas of children’s language [6], intelligence [7], self-regulation [8], and academic achievement [9]. The use of naturalistic observational designs limits conclusiveness regarding issues of causality. Indeed, genetic and unmeasured environmental confounds threaten the internal validity of observational study findings regarding issues of causal influence [10, 11]. Thus, though robust associations between responsive parenting and academic readiness have been documented, the direction of influence cannot be deciphered based on naturalistic observational studies.

One way to study the causal relations between responsive parenting and academic readiness is using randomized controlled trials (RCTs), wherein participants are randomly assigned to an intervention (e.g., parent responsiveness training) or a control group, thus balancing measured/unmeasured confounders. Post-intervention differences in children’s outcomes are attributed to the effects of the intervention, conferring a high probability for causal conclusions [12, 13]. Furthermore, mechanisms involved in interventions can be systematically examined through a mediation analysis, wherein the mediating function of parental responsiveness can be examined analytically [14].

There is a growing number of RCTs that examine whether responsive parenting interventions lead to positive changes in children’s academic readiness, with variability in methodology in terms of study design and measurement approaches [15]. With this growing knowledge base comes conflicting findings, which are difficult to interpret. Furthermore, studies have yielded inconsistent results with respect to verifying the mediating role of parental responsiveness in treatment effects on children’s developmental outcomes [14]. Therefore, a systematic synthesis of the literature and a meta-analysis is warranted at this time to estimate the magnitude of the causal effect of responsive parenting on academic readiness, and any substantive and/or methodological moderators that serve to strengthen or weaken the effect.

The proposed project will address the following objectives:

1. Obtain a precise estimate of the causal influence of responsive parenting in the infancy/early childhood period on academic readiness, including (i) problem-solving/reasoning, (ii) language, (iii) executive functioning, and (iv) pre-academic skills, respectively.
2. Examine whether substantive moderators related to developmental processes predict between-study variation in effect sizes (e.g., child age and risk status, family sociodemographics).
3. Examine whether methodological differences between studies predict variation in effect sizes (e.g., intervention characteristics, inclusion of mothers/fathers/both, publication date, outcome measurement).
4. Investigate the differential strength in the causal relationship between responsive parenting and each of the four domains of academic readiness.
5. Investigate mechanisms of change to examine if increases in responsive parenting behaviour mediate the effects of the intervention on gains in children’s skills.

Objectives 1–3 will examine each academic readiness outcome independently, via four systematic reviews and meta-analyses. Substantive and methodological moderators will be examined within each academic readiness outcome. Subsequently, objectives 4–5 will be addressed using a single meta-analysis that pools across all outcomes to investigate whether effect sizes vary as a function of academic readiness outcome, as well as to examine the mediating role of parental responsiveness in treatment effects. Pooling across outcomes for the mediation analysis will likely be necessary as we do not anticipate an adequate number of studies within each academic readiness outcome to conduct such an analysis.

## Methods

The reporting of this protocol is based on the guidance provided in the Preferred Reporting Items for Systematic Reviews and Meta-Analyses Protocols (PRISMA-P; see checklist in Additional file 1) [16] and the PRISMA 2020 statement [17]. Amendments to this protocol will be systematically tracked (with dates and rationales) and made available at the time of publication. The review was registered with the International Prospective Register of Systematic Reviews (PROSPERO; #CRD42020222143).

### Eligibility criteria

Eligibility criteria, as outlined by PICOS (population, intervention, comparators, outcomes, and study design) follow next. The population under examination is children in the prenatal, infancy, and/or early childhood period (i.e., < 6 years at baseline), and their parents. Children with special needs and/or disabilities will be excluded, including those with diagnoses of autism, intellectual/language delay, deafness/hearing loss/blindness, and/or brain injuries, as interventions including special populations typically include parenting targets that are individualized to the disorder. Interventions will have targeted responsive parenting behaviour, defined as affective-emotional responsiveness (e.g., warmth, contingent responding, acceptance), cognitive responsiveness (joint attention, rich verbal input, scaffolding), positive behavioural guidance (e.g., positive discipline, reinforcement/praise, positive behavioural support, contingencies), and/or emotion socialization (e.g., emotion coaching, validation, emotion labelling). Interventions including additional programme components (e.g., baby massage, literacy-based programmes, breastfeeding, early childhood education) will not be eligible to ensure a relatively homogeneous group of interventions (while minimising confounding programme components). Study designs will include RCTs, to minimise the risk of potential confounding factors and thus allow for an assessment of causal relations between responsive parenting and academic readiness. Comparison groups will include control groups, treatment-as-usual, and/or waitlist controls. Outcomes will include academic readiness, defined as problem-solving/reasoning, executive functioning, language, and/or pre-academics.

Language of study publication will be limited to English. Publication type is not an exclusionary criterion; published and unpublished works will be considered.

### Information source and search strategy

Studies completed from inception to the date of the search strategy will be considered. A comprehensive search of published and unpublished studies was developed by the principal investigator (first author) in consultation with librarian services, experts in knowledge synthesis research methods, and the research team (remaining authors). The following databases were included in the search, which was last executed in September 2020**:** MEDLINE (Ovid), PsycINFO (Ovid), ERIC, and ProQuest Dissertations & Theses Global. A combination of subject terms/ headings and keywords were used in the search indexing: parenting, intervention, early childhood, and academic readiness. The search strategy was developed in PsycINFO and Medline, respectively, based on common identifiers found in a set of relevant articles, and then translated to the other databases using thesaurus terms. The search strategy went through multiple iterations by testing the strategy against a set of test articles [18]. The final full electronic search strategy is available in Additional file 2. Following the full-text assessment, backward and forward searching of eligible studies will be conducted to locate additional relevant studies [19].

### Study records

#### Data management

After relevant abstract/titles are identified through the executed search strategy, citations will be downloaded in Research Information Systems (RIS) text format and imported into Covidence, a web-based software platform that streamlines the production of systematic reviews, including abstract/title screening, full-text assessment, data extraction, and risk-of-bias/quality of evidence assessments. Duplicate citations are automatically identified and removed by Covidence.

#### Selection process

To maximize interrater agreement in the selection process, we will develop standardized protocols and use calibration exercises (i.e., pilot testing) at both the title/abstract screening and full-text assessment phases. Percent agreement and kappa will be calculated and used as a guide to clarify issues and make appropriate refinements to the standardized protocol. Reviewers will start record screening/full-text assessments independently once acceptable percent agreement (i.e., 80% or more) and kappa (i.e., ≥ 0.6) are reached. Abstract/title screening will be completed by a team of four reviewers with each record screened by two independent reviewers, and discrepancies resolved by a third reviewer. Full-text assessments will be conducted by two reviewers, independently, with discrepancies resolved by a third reviewer. Study authors (first/senior) will be contacted to access full-text articles that are not available online/through library services and/or to request relevant data required for effect size extraction.

To ensure independent effect sizes within each meta-analysis, the following steps will be taken: (i) we will identify overlapping samples based on shared first/senior authorship and/or use of large-scale RCTs (e.g., Incredible Years, Triple P, Family Check Up); (ii) if there are multiple studies published using the same sample, we will select the study with objectives aligned with the current meta-analysis, the largest sample size, and psychometrically sound measurement; and (iii) studies with overlapping samples can be included if they assess different academic readiness outcomes, as these will be meta-analysed independently. Secondary analyses will pool across academic readiness outcomes, requiring independent samples across outcomes. As such, if studies include more than one academic readiness outcome, we will retain the outcome that is least represented in the meta-analysis (i.e., the outcome with the fewest number of studies).

#### Data collection process

Data will be extracted directly in Covidence. A standardized extraction protocol and form will be used for training exercises to ensure reliability in data extraction. Studies will be double coded, independently, by a team of 4–6 reviewers, with discrepancies resolved by a third reviewer.

### Data items

Data items were selected using the Cochrane Handbook as a guide, based on study objectives as well as reviewing meta-analyses in the child development and parenting literatures and selecting frequently reported data items (see Table 1).

**Table 1:** Data items for data extraction

Category	Proposed data collection
*Study methods*	
Study design	RCT Single or multicentre study; if multicentre, number of recruiting centres
Enrolment	Child age at each assessment (in months) % participants at post-intervention % participants at latest follow-up
*Participants*	
Setting	Primary care, hospital, community, home, or combination
Recruitment region	Select one of: (i) USA; (ii) UK; (iii) Canada; (iv) Australia; (v) other (specify)
Characteristics of participants	At baseline: Sample size Child sex (percentage male) Parent sex (percentage male) Parent race/ethnicity (non-minority, minority, diverse) Child race/ethnicity (non-minority, minority, diverse) Child risk status (perinatal/postnatal risk, socio-emotional/behavioural problems) Socio-demographic risk (education, income) Family-level risk status (adolescent parent, parent age, mental health)
Intervention	
Components, routes of delivery, timing, frequency, intervention protocols, length of intervention	Intervention name (list). Specific responsive parenting target (coded as affective-emotional responsiveness (e.g., warmth, contingent responding, acceptance), cognitive responsiveness (joint attention, rich verbal input, scaffolding), or positive behavioural guidance (e.g., positive discipline, reinforcement/praise, positive behavioural support, contingencies) or emotion socialization (e.g., emotion coaching, validation) Parents included, one vs. both Intensity/duration (brief description of # weeks/sessions)
Factors relevant to implementation	Delivered by (list who administered intervention)
Definition of ‘control groups’	Comparison group: coded as control; treatment-as-usual; waiting list.

### Outcomes

Primary outcomes include child academic readiness, across the four domains of academic readiness (i.e., problem-solving/reasoning, language proficiency, executive functioning, and pre-academic skills). Additionally, we will document the approach used to assess academic readiness, including direct assessment, parent-reported, and/or behavioural observation ratings.

To address multiple data points on outcomes within studies, the following steps will be taken: (i) if studies assess more than one relevant outcome across domains of academic readiness (e.g., problem-solving and language) using different measurement instruments, each will be included; (ii) if studies assess more than one relevant outcome across domains of academic readiness (e.g., problem-solving and language) using the same measurement instrument (e.g., a composite score of intelligence as well as index/subscales of specific domains), we will use the broadest measurement (i.e., composite score over index/subscale score); (iii) if studies assess more than one relevant outcome within domains of academic readiness (e.g., two measures of language), we will retain the measurement approach that is least represented in the meta-analysis; and (iv) outcome data points will be collected at baseline, post-intervention, and all follow-up assessment time points and analysed using subgroup analyses.

Secondary outcomes relate to substantive and methodological moderators of between-study variation in effect sizes, as well as assessments of parental responsiveness, which will be used to examine a pooled mediation analysis (amongst studies providing relevant data). If more than one parenting measure is available, the one that is best aligned with the study’s intervention target and has the best specificity in parenting behaviour (i.e., specific behaviours rather than global ratings) will be used. Data on parenting assessments will be subsequently coded as (i) parent-report vs. behavioural observation; and, if behavioural observations are used, (ii) micro coded vs. global/macro rating. Furthermore, an assessment will be made with respect to how well aligned the parenting assessment was with the targeted parenting behaviours in the intervention (on a scale of 0–2).

In sum, the primary question of the meta-analysis relates to the change in academic readiness outcomes that result from responsive parenting interventions. We will also examine moderators that influence the strength of effects across studies, as well as whether responsive parenting behaviour serves as a mediator of positive changes to academic readiness outcomes.

### Risk of bias in individual studies

Risk of bias will be assessed using the CLARITY Group’s tool to assess the risk of bias in RCTs [20] executed by two reviewers through discussion and consensus, covering the following types of bias: allocation, blinding, attrition, selective outcome reporting, power analysis, and baseline differences.

### Data synthesis

#### Qualitative synthesis

Summary tables will be used to present study characteristics of individual studies, including, for example, populations included (e.g., child, family, and socio-demographic risk characteristics, child age), intervention characteristics (e.g., name and comparator, nature of intervention), outcome(s) assessed (and measurement approach), and risk of bias assessment. Heterogeneity in study characteristics across studies will be described narratively and with the use of descriptive statistics (i.e., means and frequencies).

#### Quantitative analyses

The eligibility criteria were informed by a previous scoping review [15] and are expected to be restrictive enough to warrant a meta-analysis. Studies that provide sufficient quantitative data to compute an effect size will be included in the meta-analysis (further described below). Where data presented in papers is not sufficient to compute an effect size, we will contact authors to request our specific needs. Comprehensive Meta-Analysis Version 3 software [21] will be used to calculate and analyse effect sizes. To examine the overall impact of responsive parenting interventions on academic readiness domains, the standardized difference between experimental and control groups will be computed for each study (based on the means and standard deviations of each group for pre- and post-scores). All effect sizes will be calculated as Hedges’ *g* [22]. Correlations between pre- and post-test scores will be extracted to calculate the pre-post effect size [23]. Given that this is not a commonly reported statistic in this field of research [24], missing data will be addressed by contacting authors to request the necessary data. If not available, sensitivity analyses will be performed with a standardized correlation input at 0.7 vs 0.5, respectively, and results will be compared [25]. Post-intervention means and standard deviations only will be used for studies that do not provide pre-intervention data. Pooled analyses will be conducted for each academic readiness outcome, separately, using random effects modelling.

Publication bias will be examined methodologically by comparing effect sizes across published and unpublished studies via moderation analysis. Additionally, the trim-and-fill approach will be used to ensure that results are not due to test bias and to provide an indication of the reliability of reported results [26].

#### Moderation analyses

Heterogeneity of effect sizes (or, dispersion) will be assessed using the *Q* statistic. Then, the I^2^ statistic will be calculated as an estimate of true dispersion amongst study effect size estimates, i.e., the degree to which between-sample effect heterogeneity reflects true differences, as opposed to error. Finally, moderation will be assessed using Q-statistics and meta-regressions for categorical and continuous moderators, respectively [23, 27]. Primary substantive moderators will include child age at baseline, child risk factors (e.g., perinatal risks, socio-emotional/behaviour problems), and family sociodemographic risk, to examine whether the causal influence of positive parenting changes as a function of developmental age or sample characteristics. Additional methodological moderators will also be examined, as outlined in the data items table. Finally, using the integrated dataset across outcome domains (with removal of overlapping samples), we will examine whether effect sizes significantly differ across academic readiness outcomes.

#### Mediation analyses

To examine whether responsive parenting behaviour mediates the effect of parenting intervention on gains in children’s skills, we will conduct a pooled path model of mediation, with all studies that have available data [28]. We will pool effect size estimates for the following bivariate correlations provided by independent studies: (i) intervention participation and pre-post change in parenting behaviour (path a), (ii) pre-post change in parenting behaviour and pre-post change in child outcome (path b), and (iii) intervention participation and pre-post change in child outcome (path c), using independent meta-analytic approaches. Next, these pooled correlation coefficients will be used to obtain an estimate of the extent to which parenting behaviour explains the variance in the association between intervention participation and child outcome (subtracting the product of paths a*b from path c).

Finally, studies will be examined informally, on a study-by-study basis, to assess for factors that affect the presence and/or strength of a mediation effect, such as the measurement approach to parenting assessments and/or the alignment between parenting intervention targets and parenting assessments.

## Discussion

The proposed project will inform the knowledge base on the causal influence of responsive parenting on academic readiness, including whether responsive parenting has stronger links to some domains of academic readiness than others. By leveraging the strengths of RCTs and utilizing pooled mediation analyses, findings will tease apart issues of directionality. Such findings are important for furthering developmental science related to environmental contributions to academic readiness skills, an important facet of developmental health. Furthermore, findings will illuminate when and for whom responsive parenting interventions may be most effective in promoting children’s academic readiness. Such a synthesis has the potential to inform policy and practice related to minimizing social disparities in early childhood development.

Practical issues anticipated include feasibility of conducting the review in a timely matter, due to the large number of expected records to screen and full-texts to assess. Should the review go beyond 12 months after the date of initial search strategy, it will be executed again prior to publication. One practical issue that arose in the time lapse between submission of this protocol and time of revision is feasibility related to data extraction. As a result, Objective 5 was removed from the protocol at the time of data extraction (prior to data analysis). Additionally, we anticipate several effect sizes to come from overlapping samples (within or across studies). As such, caution will be taken in selecting the most appropriate outcomes a priori (as outlined in the methods).

Limitations at the study-level are anticipated with respect to risk of bias. Specifically, due to the nature of interventions included, we anticipate many, or most, studies to show a risk of bias on items related to masking of interventionists and/or participants (i.e., parents) to allocation status. That is, parents are likely to know when they are receiving a responsive parenting intervention (compared to a control condition), and interventionists are likely to know when they are delivering a responsive parenting intervention. Relatedly, it is common in developmental science to use parent-reports as a method of collecting data for outcome assessments, thus making it more likely for studies in the current systematic review to be biased on items related to masked data collection. That is, parents, who are in most cases privy to their allocation status, may also be reporting on children’s academic readiness. Some variability is anticipated to this end, wherein some studies may also include masked data collection approaches such as standardized assessment by a masked assessor. Finally, we expect study-level limitations in comprehensive data reporting including data required to compute effect sizes (e.g., pre-post correlations) and study characteristics.

Results will be disseminated through conferences presentations and publication in a peer-reviewed journal, as well as through social media outlets.

## Supplementary Information

**Additional file 1**.

**Additional file 2**.

## Data Availability

Not applicable.

## Abbreviations

RCT: Randomized controlled trials
PICOS: Population, intervention, comparators, outcomes, and study design
